# The Reform and Control of the Casualty Department

**Published:** 1907-04-13

**Authors:** 


### THE REFORM AND CONTROL OF THE CASUALTY DEPARTMENT.

### A GREAT OPPORTUNITY FOR THE BIRMINGHAM GENERAL HOSPITAL.

### Introductory.

For thirty years speakers and writers have denounced the abuse of hospitals. Many conferences have been held and more iesolutions have been passed at great meetings in London and throughout the country, but the net result has been neither progress nor effectual reform. People who are not familiar with the working of a hospital have formed the conclusion that, the majority of the patients are well-to-do people, who put off fine clothing and expensive furs and apparel in order to don humbler garb, for the purpose of visiting a voluntary hospital, founded as a charity, so as to obtain the best medical advice and any quantity of medicine there without payment. No doubt such cases have existed, and still exist, but they are relatively few, and those who know most about hospital relief have the best reasons for affirming that the abuse of hospitals in this sense is unimportant. The real cause of the existing abuse is mainly due to the abnormal increase in the number of patients who flock to the hospitals whenever they have any minor ailment or accident, however slight such lesion may in fact be.

### A Practical Remedy.

The remedy for this state of affairs is very simple. It consists in a better system for the regulation of free medical relief and its stricter enforcement. We give on another page of the present issue a model plan of a new Gate House or Admission Block, which ought to form one of the most important units in modern hospital construction. This plan is so conceived as to render it possible to deal with all the patients who may apply each day for relief at a great hospital, whilst the accommodation and arrangements are such that, every case can be treated upon its merits, and that no patient, under any circumstances, can obtain admission, either to the in- or the out-patient department, without passing through the ordeal of a preliminary examination in the hall attached to the Admission Block. It is no use for any hospital to attempt to change its system, or to regulate the numbers of those obtaining free medical relief, unless the managers are prepared to enact that, no case shall be admitted to the out-patient department except for consultative purposes, and that no patient who can properly be provided for at a free dispensary, or at the surgery of a general practitioner, shall be eprmitted to obtain free medical relief, after the first visit, in any form at the hospital.

### The Position in Birmingham.

We are glad to see from a recent speech by Mr. Neville Chamberlain, the able Chairman of the General Hospital, Birmingham, that a committee has been appointed in connection with that institution to consider this problem, and to find a solution for the many difficulties which have hitherto surrounded it. One great difficulty is that in Birmingham the General Hospital is dependent, to a large extent, for its income upon the ticket system; that is, upon a system which the public have come to regard as a means of purchasing medical relief for the poorer citizens through the contributions of the relatively well-to-do. It is held, though we cannot agree with the view, that to abolish the ticket system would mean, in fact, a large reduction in income. In other words those who know most about it maintain that there is so little of the Christian spirit underlying the gifts of the subscribers to our voluntary hospitals, that, unless they get money's-worth in the shape of relief, they will not continue to give at all, or, at least, to give adequately, for the support of the hospitals. Brought face to face with this statement, no doubt the subscribers, as a body, would declare that their motives rest on much higher ground than the low basis of a *quid pro quo*. Still, the financial question is, after all, the bed-rock on which the voluntary hospital system must rest, and it is well to avoid running any risk of diminishing the income, even temporarily, by adopting changes in administration, which might prove unpopular, until they were thoroughly mastered and understood by all those who have it in their power to give or withhold pecuniary support. We, therefore, believe that in the case of a ticket hospital it is desirable, not to issue any out-patient tickets, but to substitute for them, letters of recommendation which each subscriber can give to any poor person in whom he may be interested. When presented at the hospital, the letter will secure the bearer a thorough medical examination, though not necessarily any treatment, except, perhaps, on the first visit, unless the ailment proves to be sufficiently serious to warrant the gift of free medical relief. This simple modification would supply a remedy at the General Hospital, Birmingham, for instance, providing its committee displayed the necessary enterprise to provide, with as little delay as possible, the new unit of construction which we have entitled the Admission Block. They would thus be able to efficiently control their whole system of free medical relief.

### A Wise Expenditure Called for.

We venture to hope that the special committee which has this matter in hand at Birmingham may see its way to recommend the erection of an Admission Block similar to that of which we have given a plan this week, that they may determine to devote the out-patient department entirely to consultative purposes, and that they may not, for the sake of saving a little money, miss their present great opportunity. What intelligent Governor can be content with any makeshift because it may save a few thousand pounds of expenditure, by using portions of the existing out-patient department as a place in which casualty cases are received and dealt with. It is always bad policy to spoil the ship for a ha'p'orth of tar. Birmingham may well be proud of its great General Hospital, and that noble institution must not be rendered relatively inefficient because a few miscalled economists may hesitate to spend £5,000 or £6,000 on the erection of an Admission Block. We have satisfied ourselves that the General Hospital site is excellently well adapted for the addition of this new unit to the existing hospital buildings. We shall hope to hear that the committee have decided to put the work in hand with all reasonable despatch.

